# Intravital photoacoustic brain stimulation with high-precision

**DOI:** 10.1117/1.JBO.29.S1.S11520

**Published:** 2024-02-07

**Authors:** Guangxing Wang, Yuying Zhou, Chunhui Yu, Qiong Yang, Lin Chen, Shuting Ling, Pengyu Chen, Jiwei Xing, Huiling Wu, Qingliang Zhao

**Affiliations:** a Xiamen University, School of Public Health, Center for Molecular Imaging and Translational Medicine, Innovation Laboratory for Sciences and Technologies of Energy Materials of Fujian Province, State Key Laboratory of Vaccines for Infectious Diseases, Xiang An Biomedicine Laboratory, Xiamen, China; b Xiamen University, National Innovation Platform for Industry-Education Integration in Vaccine Research, State Key Laboratory of Molecular Vaccinology and Molecular Diagnostics, Xiamen, China; c Shenzhen Research Institute of Xiamen University, Shenzhen, China

**Keywords:** photoacoustic, brain stimulation, high-precision, non-invasive, non-genetic

## Abstract

**Significance:**

Neural regulation at high precision vitally contributes to propelling fundamental understanding in the field of neuroscience and providing innovative clinical treatment options. Recently, photoacoustic brain stimulation has emerged as a cutting-edge method for precise neuromodulation and shows great potential for clinical application.

**Aim:**

The goal of this perspective is to outline the advancements in photoacoustic brain stimulation in recent years. And, we also provide an outlook delineating several prospective paths through which this burgeoning approach may be substantively refined for augmented capability and wider implementations.

**Approach:**

First, the mechanisms of photoacoustic generation as well as the potential mechanisms of photoacoustic brain stimulation are provided and discussed. Then, the state-of-the-art achievements corresponding to this technology are reviewed. Finally, future directions for photoacoustic technology in neuromodulation are provided.

**Results:**

Intensive research endeavors have prompted substantial advancements in photoacoustic brain stimulation, illuminating the unique advantages of this modality for noninvasive and high-precision neuromodulation via a nongenetic way. It is envisaged that further technology optimization and randomized prospective clinical trials will enable a wide acceptance of photoacoustic brain stimulation in clinical practice.

**Conclusions:**

The innovative practice of photoacoustic technology serves as a multifaceted neuromodulation approach, possessing noninvasive, high-accuracy, and nongenetic characteristics. It has a great potential that could considerably enhance not only the fundamental underpinnings of neuroscience research but also its practical implementations in a clinical setting.

## 1 Introduction

In the field of neuroscience, neural stimulation has emerged as a vital approach facilitating our comprehension of how the brain functions and the management of neurological conditions. In clinical practice, electrical stimulation, employing implantable devices with metal electrodes, has demonstrated noteworthy efficacy in treating neurological disorders like Alzheimer’s disease, Parkinson’s disease, and epilepsy.^1^^–^^3^ However, the invasiveness of electrical stimulation based on metal electrodes may potentially lead to unnecessary complications, such as inflammation and bleeding.^4^ Then, noninvasive neuromodulation strategies, for example, transcranial direct current stimulation and transcranial magnetic stimulation, were developed to circumvent the need for surgical interventions.^5^^,^^6^ Yet, both of them are limited in sophisticated neural circuit manipulation due to the poor spatial (∼several millimeters).^7^^,^^8^ Recently, optogenetics harnessing light to manipulate neural activities via microbial opsins has emerged as a potent method to decipher sophisticated neural circuitries with subcellular spatial resolution and specificity in targeted cell types.^9^^–^^11^ Nonetheless, the necessity for viral transfection restricts its translation to human subjects.^12^ Considering the above-mentioned limitations, photothermal neural stimulation as a noninvasive and nongenetic neuromodulation modality has gained significant interest in fundamental neuroscience research and translational studies.^13^^–^^15^ Unfortunately, the concomitant thermal toxicity evokes a concern about potential tissue impairment.^16^ Alternatively, another novel neural manipulation technology, ultrasound neuromodulation has been employed to manipulate the neural activities in the cortex, hippocampus, and thalamus of different species, including mouse,^17^^–^^19^ monkey,^20^^–^^22^ sheep,^23^^,^^24^ and humans,^25^^–^^27^ owing to its noninvasive essence coupled with deep penetration depth.^28^^–^^30^ Despite that, during ultrasound brain stimulation, the focus and energy of acoustic wave will be compromised by skull, causing a limited spatial resolution, which fails to meet the demands of single nerve manipulation.^31^^,^^32^ Thus, there is an ongoing quest for novel neuromodulation modalities that aim to realize noninvasive, nongenetic, and high-precision neural manipulation.

**Fig. 1:**
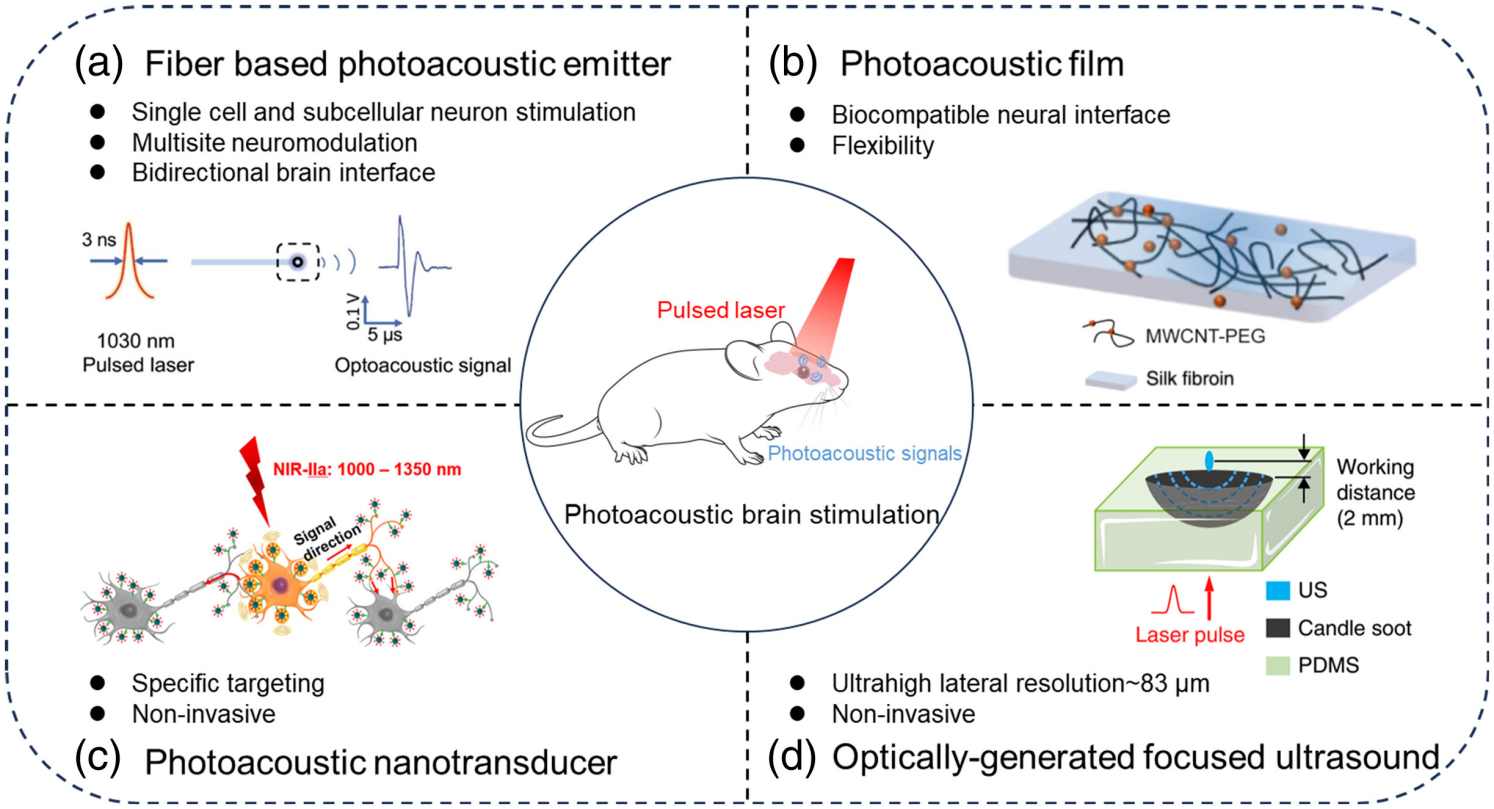
Photoacoustic brain stimulation methods. (a) Photoacoustic fiber interfaces for neural modulation.^40^^,^^44^^–^^46^ (b) Photoacoustic film neural stimulation modality based on flexible hydrogel and nanocomposite.^41^ (c) Photoacoustic neural stimulation based on nanotransducer.^42^ (d) Neural stimulation based on optically generated focused ultrasound.^43^ (Adapted with permission from Refs. 38 and 41–43.)

In recent years, the photoacoustic technique utilizes pulsed light to generate ultrasound, offering a novel alternative to traditional ultrasound technique, with high penetration depth and spatial precision, leading to rapid advancements in many fundamental and translational studies, particularly in imaging living biological structures across various scales in the life sciences.^33^^–^^37^ The photoacoustic technique was also utilized to enhance cell membrane permeability for targeted delivery of normally impermeable molecules, which further expands the capabilities of this technique.^38^^,^^39^ Particularly, in view of the high-precision, and nongenetic merits of photoacoustic technique, Jiang et al. first demonstrated the photoacoustic brain stimulation research *via* fiber-based photoacoustic emitter.^40^ Subsequently, various photoacoustic brain stimulation modalities, including photoacoustic film,^41^ photoacoustic nanotransducer,^42^ and optically generated focused ultrasound (OFUS),^43^ have been developed to further enhance the performance of photoacoustic brain stimulation and expand its application scope (Fig. 1). In brief, photoacoustic brain stimulation is a noninvasive and high-precision neuromodulation modality without genetic modification, which has great potential to open up new opportunities for basic neuroscience research and translational studies.

In this perspective, first, the mechanisms of photoacoustic generation as well as the potential mechanisms of photoacoustic brain stimulation are provided and discussed. Afterward, we summarize the current advancements of photoacoustic brain stimulation in recent years, with an emphasis on their major advances and limitations. Finally, we offer an outlook that highlights a few potential directions for further enhancing the capabilities of this emerging modality, enabling improved performance and wider-ranging applications.

## 2 Mechanisms of Photoacoustic Generation

The photoacoustic effect depends on the absorption of pulsed laser light by materials, which results in a transient temperature rise due to non-radiative relaxation processes. This temperature increase leads to a rapid thermal expansion that generates acoustic waves. Two key criteria for generating photoacoustic wave must be met, namely thermal confinement and stress confinement.^47^ By applying the concept of momentum conservation, a connection can be established between the speed at which thermoelastic expansion occurs and the amplitude of photoacoustic pressure,^48^ which can be written as

$$P_{0}≅\rho \cdot v_{s}\cdot V,$$ (1)

where ρ is the mass density ($\mathrm{kg}/m^{3}$), $v_{s}$ is the sound speed (m/s), and V is the speed of thermoelastic volume expansion. In addition, the speed of thermoelastic volume expansion (V) can be expressed as

$$V=\frac{\Delta V}{S\cdot \tau_{l}},$$ (2)

where ΔV is thermoelastic volume expansion ($m^{3}$), S is the surface area ($m^{2}$), and $\tau_{l}$ is laser pulse duration (s). The thermoelastic volume expansion (ΔV) can be defined as

$$\Delta V=\frac{A\cdot S\cdot F\cdot \beta }{\rho \cdot C_{p}},$$ (3)

where A is the light absorption (0<A<1), F is the laser fluence ($J/m^{2}$), β is volumetric thermal-expansion coefficient ($K^{-1}$), and $C_{p}$ is the specific heat capacity at constant pressure (J/kg·K). According to the Eqs. (2) and (3), the Eq. (1) can be rewritten as

$$P_{0}=\Gamma \cdot A\cdot \frac{F}{l},$$ (4)

where $\Gamma =\frac{\beta \cdot v_{s}^{2}}{C_{p}}$ is Grüneisen parameter and l is the characteristic length (m).^47^ The general consensus is that for achieving high photoacoustic amplitudes, intense light absorption and high thermal expansion are crucial.

On the other hand, to more rigorously estimate the photoacoustic amplitude, the photoacoustic equation has been described as

$$[\nabla^{2}-\frac{1}{v_{s}^{2}}\cdot \frac{\partial^{2}}{\partial t^{2}}]p(\overset{⇀}{r},t)=-\rho \cdot \beta \cdot \frac{\partial^{2}T(\overset{⇀}{r},t)}{\partial t^{2}},$$ (5)

where $p(\overset{⇀}{r},t)$ represents pressure field; $T(\vec{r},t)$ represents temperature field; and $-\rho \cdot \beta \cdot \frac{\partial^{2}T(\overset{⇀}{r},t)}{\partial t^{2}}$ is the acoustic source.^49^^,^^50^ Based on the assumption of negligible heat conduction, the time-dependent temperature field $T(\vec{r},t)$ caused by pulsed-laser heating is written as

$$\rho C_{p}\frac{\partial T(\overset{⇀}{r},t)}{\partial t}=H(\overset{⇀}{r},t),$$ (6)

where $H(\vec{r},t)$ is the volumetric nonradiative heat generation caused by light absorption ($W/m^{3}$). Thus, the Eq. (5) can be rewritten as^49^

$$[\nabla^{2}-\frac{1}{v_{s}^{2}}\cdot \frac{\partial^{2}}{\partial t^{2}}]p(\overset{⇀}{r},t)=-\frac{\beta }{C_{p}}\cdot \frac{\partial H(\overset{⇀}{r},t)}{\partial t},$$ (7)

where the heating function $H(\vec{r},t)$ can be expressed as $H(\overset{⇀}{r},t)=I\cdot f(t)\cdot g(\overset{⇀}{r})$. In this case, I is the peak intensity ($W/m^{-2}$), and f(t) and $g(\vec{r})$ are the temporal and spatial heating function, respectively. Therefore, in one-dimensional form, the photoacoustic pressure is approximately described as 49

$$p(t)∼f*g=\int f(v_{s}\tau -z)g(z)dz,$$ (8)

where τ represents the retardation time (τ=t−z/c); z denotes the distance in the z-direction; * is used to represent the convolution integral; and f and g are defined as temporal heating function and spatial light absorption function, respectively.^49^ When heat conduction is negligible during optical excitation, the convolution integral becomes an accurate tool for pressure estimation since the spatial heat source aligns with the spatial distribution of light absorption. In this case, f represents a function that varies with the duration of the laser pulse ($\tau_{l}$), and g represents a function that varies with the light absorption coefficient (α). In Eq. (8), the photoacoustic generation should be discussed in two situations: thin absorbers and thick absorbers. Assume a light pulse with a Gaussian temporal shape, labeled f(t), is projected onto thin or thick absorbers, each fully absorbing the incoming optical energy. For simplicity, the absorption profile g(z) of each absorber is uniformly distributed (similar to a rectangular function) but differs in penetration depth ($1/\alpha_{\text{thin}}$ and $1/\alpha_{\text{thick}}$ for thin and thick absorbers, respectively). Therefore, g(z) can be described as

$$g(z)=g_{0}(H(0)-H(z-1/\alpha )),$$ (9)

where is the Heaviside function ($H(z-z_{0})=0$ if $z<z_{0}$ or 1 if $z>z_{0}$), and $g_{0}$ indicates the amplitude of light absorption. In the context of light pulses with uniform fluence F, the light absorption amplitude $g_{0}$ is considerably larger for the thin absorber compared to the thick absorber, as indicated by $F=g_{0,\text{thin}}/\alpha_{\text{thin}}=g_{0,\text{thick}}/\alpha_{\text{thick}}$ Under the assumption of negligible heat conduction during the optical excitation phase, the depth of light absorption and the heat source are identical. According to the convolution integral, the heat source is effectively split into narrow segments, each producing a sound wave that has the same temporal profile as f(t). The combination of these sound waves generates the final photoacoustic wave. In the case of the thin absorber, there are fewer but higher amplitude sound waves, while the thick absorber produces more sound waves, but with lower amplitudes. Regarding the thin absorber, the photoacoustic waves formed have a high amplitude and narrow pulse width ($\tau_{l}+1/v_{s}\alpha_{\text{thin}}$). In contrast, the photoacoustic waves from the thick absorber present lower amplitudes and wider pulse width ($\tau_{l}+1/v_{s}\alpha_{\text{thick}}$). Overall, the pulse width of the photoacoustic wave is equivalent to $\tau_{l}+1/v_{s}\alpha$. Then, the characteristic length can be calculated as

$$l=v_{s}\cdot \tau_{l}+1/\alpha .$$ (10)

The photoacoustic pressure amplitude is derived by substituting the Eq. (10) into Eq. (4)

$$P_{0}=\Gamma \cdot A\cdot \frac{F}{v_{s}\cdot \tau_{l}+1/\alpha }.$$ (11)

The scenario where $v_{s}\cdot \tau_{l}$ is significantly greater than 1/α, often found in thin absorbers and expressed as $l∼v_{s}\cdot \tau_{l}$, is known as the long pulse regime. This is because the light pulse duration ($\tau_{l}$) is considerably longer than $1/v_{s}\alpha$. Conversely, in thick absorbers, when $v_{s}\cdot \tau_{l}$ is much less than 1/α (implied as l∼1/α), it’s identified as the short pulse regime. Under these circumstances, the expression for the photoacoustic pressure amplitude is rewritten as^47^

$$P_{0}=\{\begin{array}{c} \Gamma \cdot A\cdot \frac{F}{v_{s}\cdot \tau_{l}}(v_{s}\cdot \tau_{l}\gg 1/\alpha )\\ \Gamma \cdot A\cdot \frac{F}{1/\alpha }(v_{s}\cdot \tau_{l}\ll 1/\alpha ). \end{array}$$ (12)

## 3 Potential Mechanisms of Photoacoustic Brain Stimulation

**Fig. 2:**
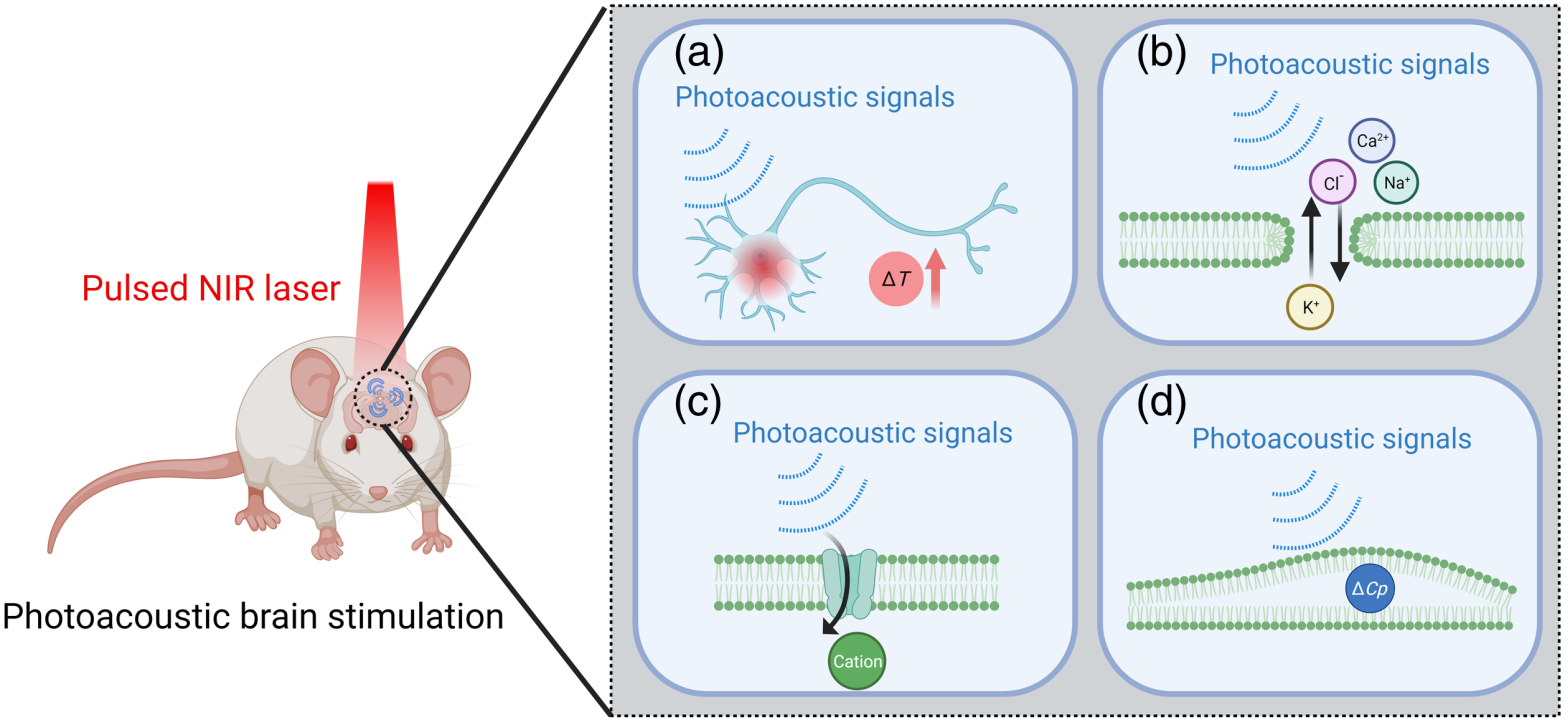
Diagram of potential mechanisms for photoacoustic brain stimulation. (a) Local temperature increases on cell membrane induced by photoacoustic effect. (b) Cell membrane pore is induced by photoacoustic effect (sonoporation), which drives ion exchange according to concentration gradients. (c) Ion channels activated by photoacoustic effect contribute to cations influx. (d) Cellular membrane capacitance (ΔCp) changes (intramembrane cavitation) are induced by photoacoustic effect.

It is important to note that while photoacoustic technique shows promise as a noninvasive and high-precision neuromodulation modality, its mechanisms are still not fully understood. In essence, photoacoustic brain stimulation utilizes pulsed laser to generate ultrasonic waves for neural stimulation. Several potential mechanisms of photoacoustic brain stimulation are provided for future research directions, including local temperature increase induced by photoacoustic effect,^51^^–^^53^ sonoporation,^54^^–^^56^ ion channel activation,^57^^–^^59^ and intramembrane cavitation,^60^^,^^61^ as shown in Fig. 2.

The phenomenon of local temperature increase on cell membrane induced by ultrasonic heating was deemed to be the fundamental mechanism with respect to the high-intensity focused ultrasound neuromodulation.^62^ Instead, investigations involving low-intensity ultrasound have revealed intriguing findings, showing only marginal temperature increases of less than 0.1°C—far below the conventional thermal threshold required for activation (ΔT>5°C).^40^ In the context of photoacoustic brain stimulation, although the employed pressures and frequencies of it fall within the spectrum of parameters utilized in ultrasound neural stimulation, it is noteworthy that photoacoustic pulses are administered with a duty cycle of 0.36%, where the heat accumulation effect is minimal.^40^ Briefly, in terms of currently developed photoacoustic brain stimulation modalities, the effect of photoacoustic heating inducing neural activities is negligible.

Another potential mechanism of photoacoustic brain stimulation is sonoporation that essentially leverages the mechanical effects of ultrasound waves to transiently and reversibly break the cellular membrane integrity, which results in ion exchange across the neural membrane and elicits neural activities. Shi et al.^38^ developed a fiber-based photoacoustic emitter, a novel ultrasound point source that overcomes the acoustic diffraction limitation. Furthermore, this emitter successfully realized the delivery of membrane-impermeable small molecules into living cells *via* the sonoporation effect, operating under a pressure of ∼40  kPa and at a low frequency. While its potential has been explored in impermeable molecule delivery, the contribution of sonoporation to altering neural activity through photoacoustic brain stimulation strategy has yet to be fully deciphered. Future investigations utilizing whole-cell electrophysiology may unlock the true contributions of sonoporation in activated neurons during photoacoustic neuromodulation.

Recently, the activation of mechanosensitive ion channels during acoustic neuromodulation has attracted widespread research interest. In *Caenorhabditis elegans*, Kubanek et al.^63^ observed MEC-4 (an ion channel required for touch sensation)-dependent currents *in vivo* during ultrasound modulation. Then, to further test whether mechanical forces can directly induce nerve cell responses, Gaub et al.^64^ demonstrated that neuronal activity can be modulated by mechanical stimuli through atomic force microscope and calcium imaging technology. Subsequently, Yoo et al.^58^ scrutinized the activation patterns of diverse mechanosensitive ion channels through ultrasound stimulation and calcium fluorescent imaging. Consequently, they discerned the pivotal involvement of three distinct ion channels: namely, TRPP2, TRPC1, and Piezo1. Nevertheless, the electrophysiological investigations at the single neuron level are limited due to the inapplicability of whole-cell recording with ultrasound stimulation. Fortunately, a tapered fiber photoacoustic emitter developed by Shi et al. is capable of stimulating single neuron or only subcellular structures, which finally makes the integration of photoacoustic stimulation with patch-clamp recording on single neuron feasible. Thus, the detailed ion channel dynamics involved in mechanical stimulation by photoacoustic neuromodulation can be further unveiled in the future.

Another prevailing explanation of how ultrasound activates neurons is intramembrane cavitation, which disturbs the structure of neural membrane and induces capacitive currents. Krasovitski et al.^61^ constructed a “bilayer sonophore” model to study how the mechanical energy of ultrasound is absorbed by the cellular membrane and induces intramembrane cavitation. The results demonstrated that the phenomenon of intramembrane cavitation was observed under the condition of continuous wave ultrasound at the frequency of 1 MHz. Then on the basis of this study, Plaksin et al.^60^ further verified that the ultrasound-induced intramembrane cavitation is capable of leading to neuron excitation *via* the effect of currents induced by changes of membrane capacitance. However, the cultured primary cortical neurons were successfully evoked by single-cycle and broad bandwidth photoacoustic wave generated by OFUS.^43^ Thus, the intramembrane cavitation mechanism may not be applicable to photoacoustic neuromodulation.

## 4 Photoacoustic Brain Stimulation Modalities

### 4.1 Fiber-Based Photoacoustic Emitters

**Fig. 3:**
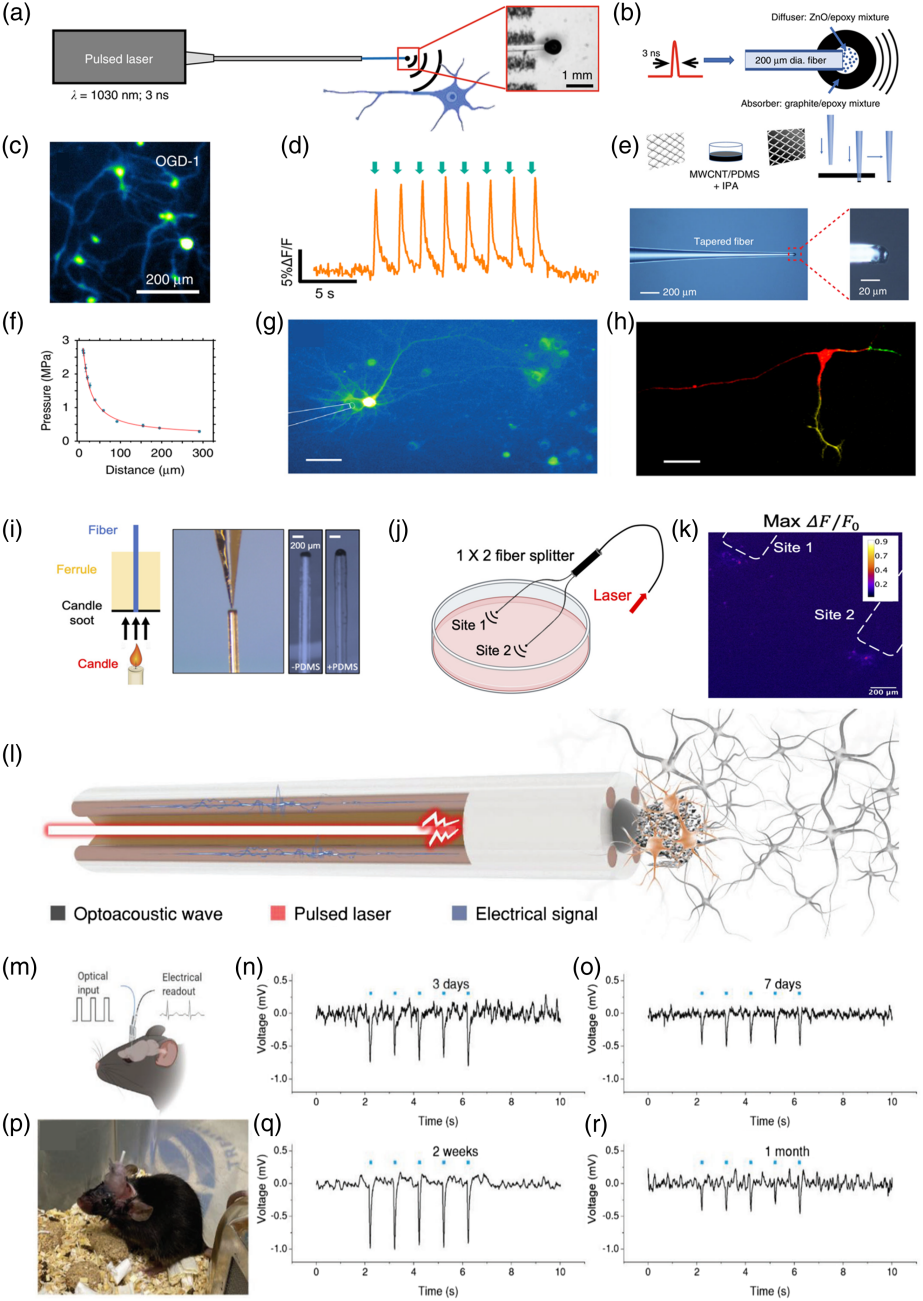
Fiber-based photoacoustic emitters. (a) The diagram of photoacoustic neuromodulation through a FOE. Inset is the enlarged FOE tip. (b) Schematic of acoustic wave generation. (c) Photoacoustic neuromodulation induced calcium transients in cultured primary neurons loaded with OGD-1. (d) Calcium trace of a neuron undergone repeated FOE stimulation. Green arrow: stimulation onset. (Adapted with permission from Ref. 40.) (e) Multiwall CNT/PDMS mixture as coating material casted on a metal mesh followed by a punch-through method to coat the tapered fiber. (f) Detected pressure plotted as a function of the distance. (g) TFOE-induced stimulation of GCaMP6f expressing single neuron. Scale bar: 50  μm. (h) TFOE selectively stimulation of axon (red) and dendrites (yellow and green) of a multipolar neuron. Scale bar: 50  μm. (Adapted with permission from Ref. 44.) (i) Key steps of CSFOE fabrication. Scale bars: 200 mm. (j) Diagram of dual site stimulation using two CSFOEs with a fiber splitter. (k) Map of the max $\Delta F/F_{0}$ image of two sites of neurons stimulated by two CSFOE. (Adapted with permission from Ref. 45.) (l) Diagram of mFOE for bidirectional communication with neurons. (m) Illustration of the mFOE enabled bidirectional neural communication using laser signal as input and electrical signal as readout. (p) mFOE was implanted into hippocampus of a wild type C57BL/6J mouse. Simultaneous optoacoustic stimulation and electrophysiological recording performed at 3 days (n), 7 days (o), 2 weeks (q), and 1 month (r) after implantation. (Adapted with permission from Ref. 46.)

Lately, fiber-based photoacoustic emitters were developed as a miniature ultrasound source for all-optical ultrasound imaging and surgical guidance.^65^^,^^66^ Beyond these applications, Jiang et al.^40^ first demonstrated the innovative application of fiber-based photoacoustic emitter modality in neuromodulation at submillimeter spatial precision [Figs. 3(a) and 3(b)]. The fiber-based photoacoustic emitter termed as fiber-optoacoustic converter (FOC) fabricated by coating a fiber tip with a light diffusion layer (ZnO/epoxy mixture) and an absorption layer (graphite/epoxy mixture) in this study has a diameter of 600  μm and can activate neurons within a radius of 500  μm, providing superior spatial resolution compared to conventional ultrasound neuromodulation. Calcium transients were observed in response to laser pulse trains delivered by the FOC, and no morphological changes were detected in the stimulated neurons [Figs. 3(c) and 3(d)]. Additionally, the FOC was able to evoke motor responses with high spatial precision in the motor cortex. No response was observed in the contralateral A1, indicating that the auditory pathway was not involved in the neural activation. In brief, the FOC has shown promising potential for high-precision neural stimulation without the need for genetic modification.

To further improve the spatial precision of photoacoustic stimulation, Shi et al.^44^ proposed a further miniaturized fiber-based photoacoustic emitter termed as tapered fiber optoacoustic emitter (TFOE), which is capable of manipulating a single neuron with an unprecedented high spatial precision [Figs. 3(e)–3(h)]. The researchers fabricated the TFOE with a diameter of 20 μm at the tip [Fig. 3(e)], in which the absorption/thermal expansion layer using carbon nanotubes (CNTs) embedded in polydimethylsiloxane (PDMS) was optimized to improve photoacoustic conversion efficiency. The spatial resolution of the acoustic field generated by TFOE was found to be 39.6  μm [Fig. 3(f)], satisfying single-cell manipulation. Importantly, TFOE stimulation successfully targeted subcellular structures, such as axons and dendrites, within neurons [Figs. 3(g) and 3(h)]. Thus, these results revealed that TFOE has neurostimulation capabilities with high accuracy and reliability, providing new possibilities for neuroscience studies at the level of individual neurons and potential clinical applications without genetic modifications.

Generally, multiple functional regions of the brain are always involved in sophisticated brain functions. Thus, a high-precision and multi-site stimulation tool is needed. For achieving this goal, based on their previous study,^40^ Chen et al.^45^ developed a new fiber-based photoacoustic emitter named as candle soot-based fiber optoacoustic emitters (CSFOE) with high photoacoustic conversion efficiency [Figs. 3(i)–3(k)]. The CSFOE was fabricated by coating the tip of a polished multimode optical fiber with candle soot synthesized from a paraffin wax candle, followed by coating with PDMS using a nanoinjector [Fig. 3(i)]. Besides, the pressure of the generated acoustic signal by CSFOE reached approximately 10 MPa, which is 9.6 times larger compared with that generated by FOC. Based on this advantage, CSFOE successfully realized dual-site neuron stimulation with an average maximum fluorescence change of over 10% in GCaMP6f-labeled neuron cultures [Figs. 3(j) and 3(k)]. Therefore, the CSFOE had superior spatial resolution and high-pressure conversion efficiency, making it suitable for modulating complex animal behavior by controlling multiple target sites in the brain circuitry.

Bidirectional communication with neural circuits in the brain is crucial for fundamental studies and clinical treatments of neurological diseases. However, existing methods such as electrical stimulation and optogenetics have limitations in terms of interference with electrical recording, low efficiency in viral transfection, and safety concerns, respectively. To overcome the limitations of existing methods, Zheng et al.^46^ developed a multifunctional fiber-based optoacoustic emitter (mFOE) that combines photoacoustic neuromodulation and electrical recording [Fig. 3(l)], which is orthogonal to electrical recording and does not require viral transfection, making it a promising candidate for bidirectional brain interfaces [Figs. 3(l)–3(r)].

### 4.2 Photoacoustic Film

**Fig. 4:**
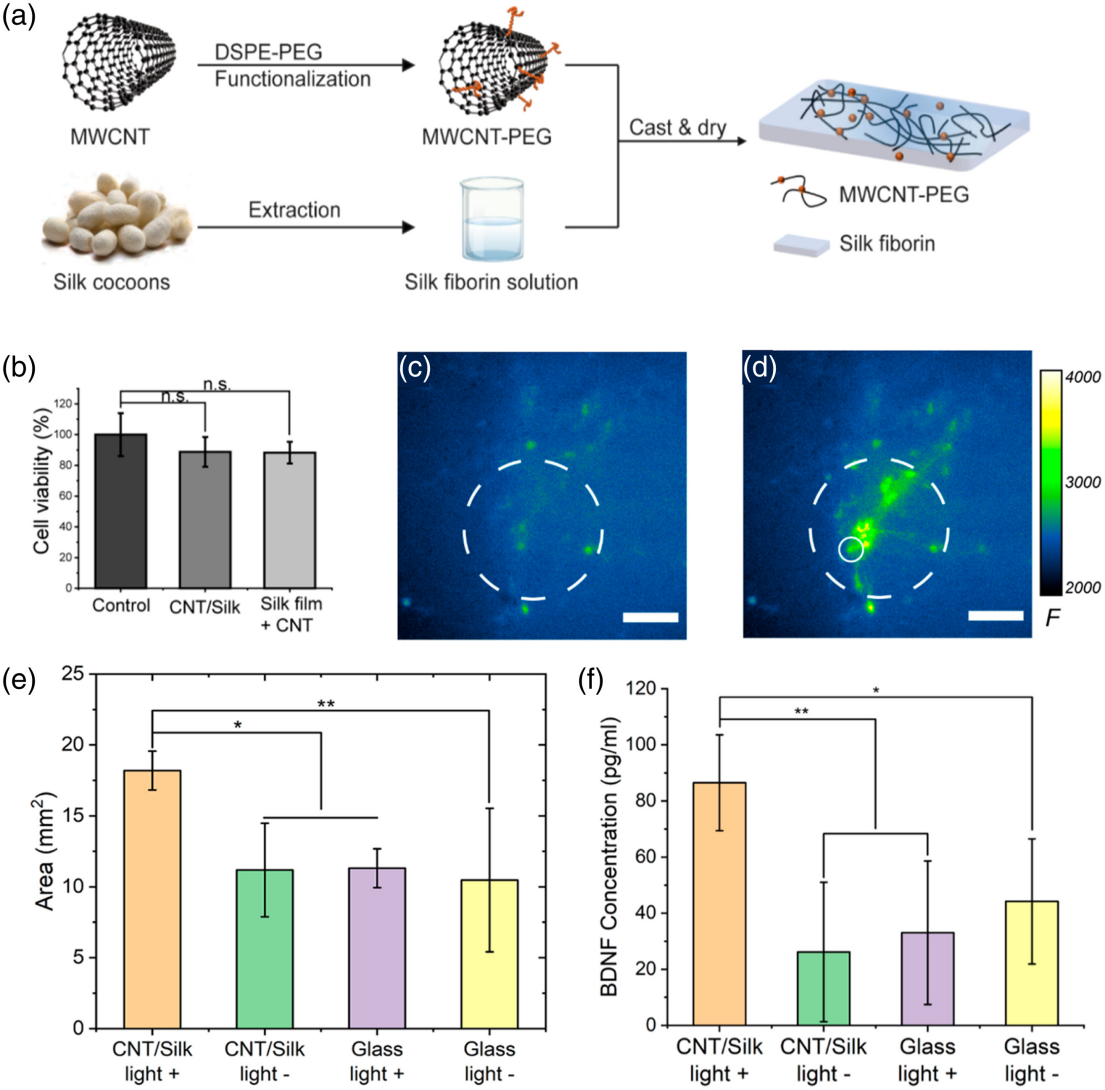
Flexible and biocompatible photoacoustic film for neural stimulation and regeneration. (a) Diagram of the fabrication process of photoacoustic film. (b) Biological safety of silk film (as control), CNT/silk film, and silk film with freeform CNT. Calcium images of rat cortical neurons (d) before and (c) after photoacoustic neural stimulation. (e) Average neurite coverage area for dorsal root ganglion cells in four groups. (f) Average concentrations of BDNF of photoacoustic stimulated and unstimulated dorsal root ganglion cells. (Adapted with permission from Ref. 41.)

Utilizing biocompatible scaffolds as neural interfaces is crucial for the functional repair of nerve injuries and rehabilitation of neurodegenerative diseases. Furthermore, neural stimulation has been found to promote neural regeneration.^67^ Therefore, various factors such as mechanical^68^ and chemical stimuli^69^ were involved in functionalizing nerve scaffolds, in which electrical stimulation^70^^,^^71^ is the most widely applied technique. However, the delivery of electrical stimulus to conductive scaffolds remains challenging, and current solutions have limitations in terms of spatial resolution and the risk of infection. Aiming to overcome these limitations, Zheng et al.^41^ proposed a novel photoacoustic neural stimulation modality, a flexible and biocompatible photoacoustic film, for promoting neural regeneration (Fig. 4). This photoacoustic film was fabricated by embedding functioned CNTs, efficient photoacoustic agent, into silk fibroin solution, an FDA-approved biocompatible material, and casting the mixture [Fig. 4(a)]. The viability of cortical neurons cultured on photoacoustic film was evaluated using the MTS assay, and no significant difference in cell viability was observed compared to the control group [Fig. 4(b)]. Neurons cultured on photoacoustic film showed increased fluorescence intensity after photoacoustic stimulation [Figs. 4(c) and 4(d)]. Furthermore, by promoting the secretion of brain-derived neurotrophic factors (BDNF), this innovative photoacoustic stimulation modality has been proven capable of facilitating neural regeneration [Figs. 4(e) and 4(f)]. Compared with other neuromodulation modalities, such as electrical stimulation and optogenetics, this innovative photoacoustic film eliminates the need for cumbersome wire connections and genetic modifications, making it a convenient and versatile option for researchers and clinicians. Moreover, photoacoustic film is complementary to other photoacoustic brain stimulation modalities like fiber-based photoacoustic emitters, further expanding its potential applications in the field of neural stimulation.

### 4.3 Photoacoustic Nanotransducer

**Fig. 5:**
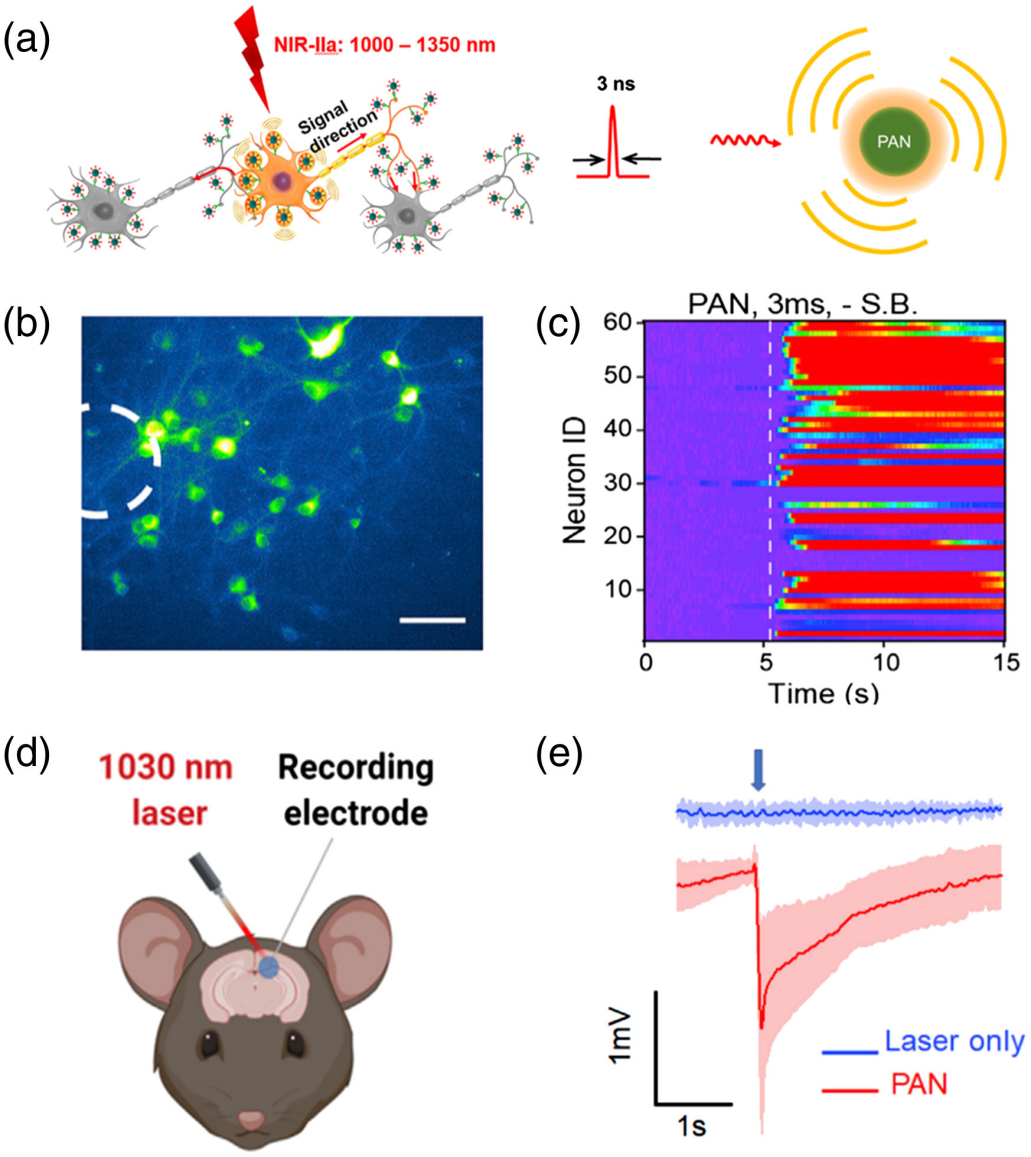
Nanotransducer-mediated photoacoustic brain stimulation. (a) Diagram of photoacoustic nanotransducer induced neural stimulation (left) and the PAN generating photoacoustic signal generated by illuminating photoacoustic nanotransducer with nanosecond laser pulses (right). (b) Calcium images of neurons transfected by GCaMP6f and cultured with photoacoustic nanotransducer for 15 min. White circle: illumination position. (c) Colormaps of fluorescence changes of neurons stimulated by photoacoustic nanotransducer. (d) Diagram of *in vivo* neural stimulation by injected photoacoustic nanotransducer coupled with electrophysiology measurement. (e) Electrophysiology curves recorded at the brain region without photoacoustic nanotransducer as the control (blue) and photoacoustic nanotransducer treated region (red). Blue arrow: stimulation onset. (Adapted with permission from Ref. 42.)

Recently, there has been a remarkable surge in the development of nanoparticle-assisted neuromodulation techniques.^72^^–^^74^ Particularly, semiconducting polymer nanoparticles exhibit unique advantages, such as the remarkable ability to absorb near-infrared light, ensuring optimal biocompatibility, and allowing for controlled biodegradation.^75^ Building upon this, Jiang et al.^42^ innovatively developed a photoacoustic brain stimulation nanocomposite platform, termed as photoacoustic nanotransducer, which was created using bis-isoindigo-based polymer (BTII) and modified with poly(styrene)-b-poly(acrylic acid) (PS-b-PAA) forming water-soluble nanoparticles (∼50  nm) *via* nanoprecipitation (Fig. 5). *In vitro* experiments were successfully conducted to activate the primary neurons using nanosecond laser pulses at 1030 nm [Figs. 5(b) and 5(c)], which have been verified that the high temporal resolution (∼ms) and single-cell spatial resolution can be realized. Besides, the stimulation specificity of photoacoustic nanotransducer was further achieved by conjugating the mechanosensitive ion channel TRPV4 antibody with photoacoustic nanotransducer to target the mechanosensitive TRPV4 channels on the neuronal membrane. Furthermore, *in vivo* experiments were performed by injecting photoacoustic nanotransducer directly into the brain to activate the motor cortex and subsequent motor responses were observed via electromyography (EMG) recordings. Briefly, harnessing NIR-II excitation, the nongenetic neuromodulation modality is capable of achieving deep tissue penetration and targeting cellular specificity stimulation.

### 4.4 Optically Generated Focused Ultrasound by Curved Soft Optoacoustic Pad

**Fig. 6:**
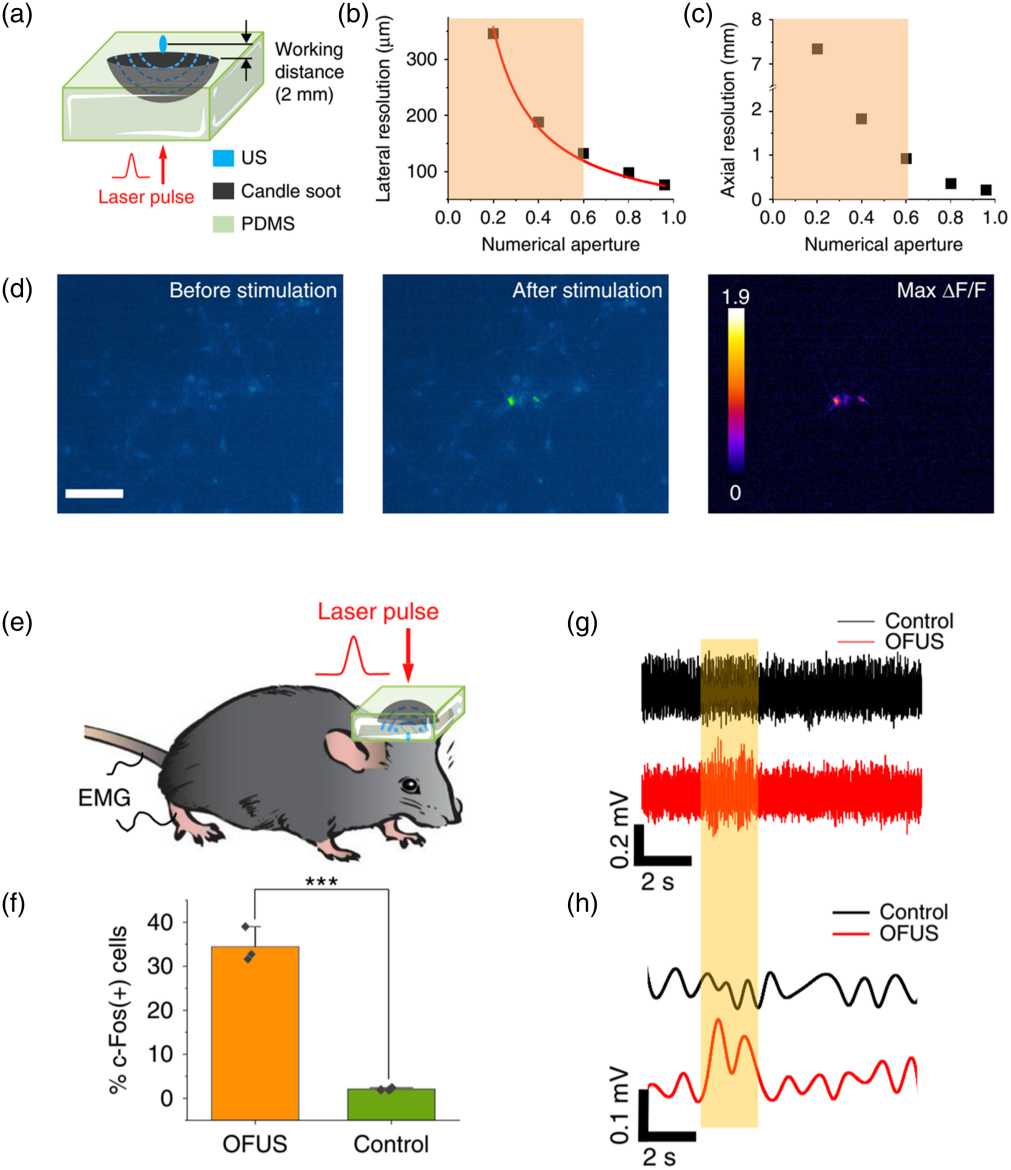
OFUS for neuromodulation. (a) The diagram of OFUS design. Numerical aperture and lateral resolutions (b) and numerical aperture and axial resolutions (c). Orange area: the NA range of conventional ultrasound transducers. (d) Calcium images of neuron activities before and after OFUS stimulation. Scale bar: 50  μm. (e) The diagram of OFUS *in vivo.* (f) Statistic analysis of the percentage of c-Fos positive neurons after OFUS stimulation. (g) EMG recordings of 2 s OFUS stimulation at the somatosensory cortex. Orange box: laser on. (h) EMG signals after the band-pass filter and full-wave rectifier and envelope. (Adapted with permission from Ref. 43.)

While previously developed fiber-based photoacoustic emitters neuromodulation modalities are capable of achieving non-genetic, high-precision neural stimulation, a surgical implantation procedure is usually required because the fiber-based photoacoustic emitters utilize near-field ultrasound for localized neural stimulation. To overcome this limitation, Li et al.^43^ proposed a novel photoacoustic brain stimulation modality called OFUS for noninvasive brain stimulation with ultrahigh precision (Fig. 6). In this study, the OFUS was generated by a curved soft optoacoustic pad (SOAP), fabricated by candle soot layered with PDMS, upon a pulsed laser illumination [Fig. 6(a)]. OFUS produced an ultrahigh spatial resolution of approximately 83  μm, significantly better than transcranial-focused ultrasound (tFUS). Upon the illumination of a pulsed laser, OFUS successfully evoked neuron excitation observed by calcium imaging *in vitro*, which demonstrated that OFUS has the ability to evoke responses in neurons and achieve localized stimulation [Fig. 6(b)]. Additionally, the transcranial stimulation capability of OFUS generated by SOAP was also investigated *in vivo*. The stimulation results were evaluated by both immunofluorescence imaging (c-Fos) and electrophysiology recording [Figs. 6(e)–6(h)]. Significantly, the c-Fos signal remained localized exclusively within the designated target site, encompassing an approximate area of 200  μm in diameter. This outcome effectively showcases a superior spatial resolution compared to the conventional tFUS stimulation (1∼5  mm).^76^ Collectively, OFUS provides exceptionally precise non-invasive methodologies for delving into neurological investigations within the sub-regions of the brain, which has great potential to be a crucial technology for advancing both neuroscience research and clinical interventions.

## 5 Outlook and Future Directions

As previously deliberated, photoacoustic brain stimulation emerging as a novel and multifaceted modality has posed great potential to propel the domain of acoustic neuromodulation forward, spanning not only fundamental scientific exploration but also intricate clinical utilizations. While utilizing the emerging photoacoustic neuromodulation approach represents a promising advancement in addressing prior challenges, its current stage reveals limitations that underline the need for ongoing improvements in the field. Aiming to achieve high-precision and non-genetic neuromodulation, fiber-based photoacoustic emitters photoacoustic stimulation modalities including FOC, TFOE, CSFOE, and mFOE were developed and characterized by single neuron stimulation, sub-cellular stimulation, multi-site stimulation, and bidirectional communications, respectively. Unfortunately, these modalities require surgical implantation into the target area and are not suitable for transcranial application. Besides, the future development direction of fiber-optic stimulators is heading towards multifunctionality. Thus, in future work, one can combine pharmacological intervention channel with this fiber-based photoacoustic stimulation modality to construct a multifunctional fiber-based photoacoustic emitter for achieving more sophisticated brain research. In addition, to tackle the problem of different form of neural interface requirements in neuromodulation areas such as the brain cortex, retina, and peripheral nerve, a flexible and biocompatible scaffold with photoacoustic properties was developed. The photoacoustic film, when excited with a 1030 nm pulsed laser, produced a broadband photoacoustic wave, initiating a calcium influx in neurons and facilitating in the proliferation of neurites. Nevertheless, the process of implanting photoacoustic film is still invasive and might not be practical for larger-volume condition. Therefore, using injectable materials could be a potential answer to address this clinical challenge.^77^ Moreover, photoacoustic nanotransducer, a new nongenetic nanoparticle-assisted neuromodulation platform, was created to achieve neural activation with enhanced specificity by conjugating with TRPV4 antibody. The functionality of photoacoustic nanotransducer for neuromodulation in a living organism was confirmed by its direct administration into the motor cortex of a mouse and triggering it with an NIR II laser light. However, the delivery process of photoacoustic nanotransducer was invasive. And, the penetration depth of NIR II laser light utilized in this study was limited in deep cerebral nuclei stimulation. New photoacoustic nanotransducer, conjugating with specific neural cell membrane channel protein antibody, with capabilities of penetrating blood brain barrier, or noninvasive blood brain barrier opening delivery method, and advanced optical wavefront shaping method for deep tissue focus should be developed to realize cell-specific, nongenetic and noninvasive photoacoustic nanotransducer neuromodulation in future. Furthermore, as an exogenous agent, the potential toxicity of photoacoustic nanotransducer introduced into animals and eventually in patients should be further confirmed in subsequent research. On the other hand, to realize high-precision photoacoustic neuromodulation without any surgical implantation, the OFUS generated by SOAP was created, which can perform high-precision transcranial neuromodulation compared with traditional ultrasound stimulation. However, the spatial resolution of SOAP is inferior to that of fiber-based photoacoustic emitters. That is caused by the distortion of thick skulls. Therefore, in future work, acoustic wavefront engineering should be done to compensate for this aberration. In summary, different photoacoustic neuromodulation platforms possess distinct characteristics and advantages. Fiber-based photoacoustic emitter modalities, such as TFOE and mFOE, exhibit strengths in the field of single cell/subcellular stimulation and bidirectional communication (i.e., carrying out both neural stimulation and simultaneous electrical recording of neural responses). With its outstanding biocompatibility and flexibility, the photoacoustic film is adept at forming conformal attachments to tissues with varying shapes. Moreover, the photoacoustic nanotransducer, integrated with an antibody coupling strategy, opens up possibilities for precisely targeted stimulation of specific cell type. In addition, OFUS is capable of performing noninvasive and ultrahigh precision (below 0.1 mm) neuromodulation without surgical implantation, proving to be an essential technology in the fields of neuroscience research and clinical therapy. The pros and cons of four different photoacoustic neuromodulation platforms are shown in Table 1.

**Table 1:** Pros and cons of four different photoacoustic neuromodulation platforms.

Platform	Pros	Cons
Fiber-based photoacoustic emitters	Single cell/subcellular precision, and bidirectional communication	Invasive
Photoacoustic film	Biocompatible and flexible	Implantation process remains invasive
Photoacoustic nanotransducer	Specific targeting	Invasive delivery, limited depth, and biosafety should be further confirmed
OFUS	Non-invasive transcranial neural stimulation with ultrahigh precision (below 0.1 mm)	Skull distortion

OFUS: optically generated focused ultrasound.

Ensuring the brain’s safety with photoacoustic wave is vital for the feasibility of photoacoustic neuromodulation as an effective brain stimulation technique. Mechanical and thermal effects are the main safety concerns during photoacoustic stimulation. First, the mechanical index (MI), a non-dimensional measure widely employed in the field of ultrasound, serves to estimate the potential for mechanical damage caused by ultrasound. It is represented as^52^

$$MI=\frac{P_{\text{negative}}}{\sqrt{F_{c}}},$$ (13)

where $P_{\text{negative}}$ is peak negative pressure of the ultrasound wave (MPa) and $F_{c}$ is the center frequency of the ultrasound pulse (MHz). In the case of mFOE and OFUS, the MI of generated photoacoustic wave is 0.2 and 0.5, respectively, which is below 1.9, the recommended safety limit set by the FDA guidelines.^46^^,^^78^ Moreover, the peak negative pressure of FOC, TFOE, CSFOE, photoacoustic film, photoacoustic nanotransducer was estimated below the threshold of bubble cloud generation in soft tissue (25 to 30 MPa).^79^

For thermal safety, in FOC, TFOE, CSFOE, mFOE, photoacoustic film, OFUS platform, the maximum temperature increase does not exceed 1.6°C. Such temperature increase is well below the previously reported threshold for thermal induced neural damage.^80^ However, in photoacoustic nanotransducer platform, a rapid temperature surge was simulated, peaking at 8.4°C at the photoacoustic nanotransducer surface and attaining 5.0°C 10 nm away from the surface, which may introduce thermotoxicity during chronic *in vivo* stimulation under body temperature. Further thermal safety research is needed in future. These initial studies are promising, but there is a need for more extensive studies to fully determine the safety and efficacy of photoacoustic neuromodulation for immediate applications in the brain, and also to set safety guidelines for upcoming chronic applications.

Different frequencies used in ultrasound stimulation produce distinct results due to its frequency-specific nature. For example, prior research^81^ has demonstrated variability in the effectiveness of neuron spiking induction by ultrasound neurostimulation in mice, across a frequency spectrum of 0.3 to 2.9 MHz. Higher frequencies in this range require increased spatial peak intensities to maintain the same effectiveness as lower frequencies. In addition, neural inhibition effects were induced using high frequency ultrasound operating at 30 MHz.^82^ Nevertheless, due to the broad bandwidth nature of the photoacoustic wave, currently, it is limited to isolate frequency-specific responses during photoacoustic neuromodulation. So far, no studies have shown that photoacoustic neuromodulation technology can hyperpolarize neurons to inhibit their activity. Notably, brain network connectivity and activities are highly complex, and stimulating or inhibiting these neuronal activities is vital to comprehend their functions. Limitation of isolating frequency-specific responses and absent the capacity to suppress the activity of neurons impeded the study of complex neural activities by photoacoustic neuromodulation technology. On the other hand, the advantage of photoacoustic broadband stimulation relative to specific frequency ultrasound stimulation is that photoacoustic neuromodulation can stimulate neuronal activity using acoustic waves with shorter pulse duration (∼1  μs) than those used in ultrasound stimulation. Therefore, the relative advantages and disadvantages of broadband stimulation in photoacoustic stimulation should be carefully considered depending on the specific application and research goals. Additionally, to date, there is no clear understanding about the mechanism of photoacoustic stimulation. Fortunately, TFOE is metal-free and compatible with patch clamp recording technology. Thus, future investigations utilizing TFOE, electrophysiological recording, genetic and pharmacological intervention could provide insight into the molecular mechanism of photoacoustic stimulation.

## Data Availability

Data sharing is not applicable to this article, as no new data were created.
